# Casos graves de hepatite A: série de casos em Curitiba, Paraná, 2023-2024

**DOI:** 10.1590/S2237-96222026v35e20250869.pt

**Published:** 2026-03-27

**Authors:** Janilza Silveira Silva, Renata Barbosa Vilaça Marques de Carvalho, Alcides Souto de Oliveira, Liza Regina Bueno Rosso, Leia Regina Silva, Diego Spinoza dos Santos, Clea Elisa Ribeiro, Katiuska Ferraz Jansen Negrello, Cláudia Weingaertner Palm, Dirlene Pacheco Venâncio, Monique Boese, Aroldo José Borges Carneiro, Tatiane Bartneck Telles, Silvio Luis Rodrigues Almeida

**Affiliations:** 1 Ministério da Saúde, Departamento de Emergências em Saúde Pública, Brasília, DF, Brasil; 2 Centro de Epidemiologia da Secretaria Municipal da Saúde de Curitiba, PR, Brasil; 3 Centro de Informações Estratégicas em Vigilância em Saúde de Curitiba, PR, Brasil; 4 Prefeitura de Curitiba, Secretaria Municipal de Saúde, Curitiba, PR, Brasil

**Keywords:** Hepatite, Vírus da Hepatite A, Insuficiência Hepática Aguda, Falência Hepática, Estudos Descritivos., Hepatitis, Virus de la Hepatitis A, Fallo hepático agudo, Fallo Hepático, Estudios Descriptivos.

## Abstract

**Objetivo:**

Descrever o perfil epidemiológico do surto de hepatite A em Curitiba, Paraná, entre novembro de 2023 e maio de 2024, com ênfase na caracterização clínica e laboratorial dos casos que evoluíram para óbito ou transplante hepático.

**Métodos:**

Estudo descritivo tipo série de casos baseado em casos confirmados de hepatite A. Casos graves, definidos por óbito ou transplante hepático, foram identificados via Sistema de Informação sobre Mortalidade e Núcleos Hospitalares de Vigilância Epidemiológica. Dados clínico-laboratoriais foram obtidos por revisão de prontuários. Calcularam-se proporções, medidas de tendência central e dispersão.

**Resultados:**

Entre os 281 casos confirmados, a maioria era do sexo masculino (74,7%), com idade mediana de 29 anos. Seis pessoas (2,1%) evoluíram para formas graves, dos quais três receberam diagnóstico de hepatite fulminante. A mediana de idade dos casos graves foi de 43 anos, predominância masculina (5/6) e dois eram pessoas em situação de rua. Entre os casos graves, cinco foram óbitos e um transplante hepático. Os sintomas e complicações mais comuns foram icterícia, insuficiência renal aguda e necessidade de ventilação mecânica (5/6). Resultados laboratoriais indicaram disfunção hepática (transaminase glutâmico-oxalacética mediana de 3.448 U/L; razão normalizada internacional mediana de 3) e disfunção renal (mediana de ureia 52 mg/dL e de creatinina 3 mg/dL). A mediana de hospitalização foi de 11 dias.

**Conclusão:**

Nesse surto ocorreram casos com necessidade de transplante hepático e evolução para óbito, evidenciando que, embora geralmente autolimitada, a doença pode apresentar evolução grave. A resposta oportuna e a vacinação direcionada podem ser estratégias-chave em cenários de surto.

## Resumen

**Objetivo:**

Describir el perfil epidemiológico del brote de hepatitis A en Curitiba, Paraná (Brasil), entre noviembre de 2023 y mayo de 2024, con énfasis en la caracterización clínica y de laboratorio de los casos que evolucionaron a muerte o a trasplante hepático.

**Métodos:**

Estudio descriptivo, de tipo serie de casos, basado en casos confirmados de hepatitis A. Los casos graves definidos por muerte o trasplante hepático se identificaron en el Sistema de Información de Mortalidad y en los Centros de Vigilancia Epidemiológica Hospitalaria. Los datos clínicos y de laboratorio se obtuvieron mediante una búsqueda en los registros médicos. Se calcularon proporciones, medidas de tendencia central y dispersión.

**Resultados:**

Entre los 281 casos confirmados, la mayoría fueron hombres (74,7%), con una mediana de edad de 29 años. Seis personas (2,1%) progresaron a cuadros graves, de las cuales tres tuvieron diagnóstico de hepatitis fulminante. La mediana de edad de los casos graves fue de 43 años, con predominio de hombres (5/6), y dos casos eran de personas sin hogar. Entre los casos graves, cinco fueron muertes y un trasplante de hígado. Los síntomas y las complicaciones más comunes fueron ictericia, insuficiencia renal aguda y necesidad de ventilación mecánica (5/6). Los resultados de laboratorio indicaron disfunción hepática (mediana de transaminasa glutámico-oxalacética de 3.448 U/L; mediana de la razón internacional normalizada de 3) y disfunción renal (mediana de urea 52 mg/dL y creatinina 3 mg/dL). La estancia hospitalaria media fue de 11 días.

**Conclusión:**

En este brote, hubo casos que requirieron trasplante hepático y que tuvieron progresión a muerte, lo que demuestra que, aunque generalmente autolimitada, la enfermedad puede presentar una evolución severa. La respuesta oportuna y la vacunación adecuada pueden ser estrategias clave en los escenarios de brotes.

**Aspectos éticos**

**Esta pesquisa respeitou os princípios éticos, obtendo os seguintes dados de aprovação:**

Comitê de ética em pesquisa

Comissão Nacional de Ética em Pesquisa 

Número do parecer

7.545.013 

Data de aprovação

13/6/2025

Certificado de apresentação de apreciação ética

87956525.0.0000.0008

Registro do consentimento livre e esclarecido

Não se aplica.

## Introdução

A hepatite A é uma doença infecciosa aguda do fígado, causada pelo vírus da hepatite A, um picornavírus de ácido ribonucleico não envelopado pertencente ao gênero *Hepatovirus* (1,2). Sua transmissão ocorre principalmente pela via fecal-oral, por meio do consumo de água ou alimentos contaminados, bem como pelo contato direto (oro-anal) com pessoas infectadas (3).

Embora a maioria dos casos seja autolimitada, com recuperação espontânea especialmente entre crianças, a infecção pode evoluir para formas graves, sobretudo em adultos ou em indivíduos com comorbidades (4). Nesses casos, podem ocorrer hepatite fulminante - caracterizada por insuficiência hepática aguda, encefalopatia e coagulopatia - além de complicações sistêmicas como insuficiência renal aguda, sepse e distúrbios metabólicos, muitas vezes exigindo cuidados intensivos e transplante hepático como medida de resgate (2).

Nas últimas décadas, observa-se em diversos países uma transição no padrão epidemiológico da hepatite A (5). A melhoria nas condições de saneamento reduziu a exposição precoce ao vírus, aumentando a suscetibilidade entre adolescentes e adultos jovens, faixas etárias nas quais a infecção pode ser mais sintomática e grave (5). No Brasil, essa transição também se manifesta com surtos localizados, muitas vezes afetando populações específicas, como homens que fazem sexo com homens (6).

Entre novembro de 2023 e maio de 2024, o município de Curitiba, Paraná, Brasil, vivenciou um surto de hepatite A com ocorrência de óbitos (7). A circulação viral da hepatite A em surtos localizados, como o registrado em Curitiba, impõe um risco significativo à população adulta, que é mais suscetível a formas graves da doença (8) . A ocorrência potencial de desfechos clínicos desfavoráveis, como a hepatite fulminante, exige o reconhecimento precoce de fatores de risco clínico e epidemiológico para mitigar a mortalidade. A caracterização dos casos graves é, portanto, essencial para subsidiar a vigilância, otimizar o manejo clínico e fortalecer a resposta em saúde pública. 

O estudo teve como objetivos descrever o perfil epidemiológico do surto de hepatite A em Curitiba, Paraná, entre novembro de 2023 e maio de 2024, com ênfase na caracterização clínica e laboratorial dos casos que evoluíram para óbito ou transplante hepático.

## Métodos

### Delineamento

Trata-se de estudo descritivo tipo série de casos de hepatite A, com ênfase nos desfechos de gravidade, confirmados em Curitiba, Paraná, Brasil, entre 1º de novembro de 2023 e 29 de maio de 2024.

### Contexto

Curitiba, Paraná, possui uma população residente de 1.773.718 habitantes (9). Este estudo foi conduzido no contexto de um surto de hepatite A que levou à notificação de 281 casos confirmados, abrangendo o período de 1º de novembro de 2023 a 29 de maio de 2024. O sistema municipal de vigilância epidemiológica realiza a notificação de hepatite A no Sistema de Informação de Agravos de Notificação (Sinan) e o monitoramento da gravidade do surto foi verificado com dados do Sistema de Informação sobre Mortalidade (SIM) e articulação com os Núcleos Hospitalares de Vigilância Epidemiológica (NHVE). 

Este estudo foi conduzido por uma equipe do Programa de Treinamento em Epidemiologia Aplicada aos Serviços do Sistema Único de Saúde (EpiSUS-Avançado), em parceria com a equipe de vigilância local, de modo que os achados pudessem ser prontamente aplicados na resposta ao surto. Ele se originou de uma ação de vigilância epidemiológica urgente a convite do município.

### Participantes

O estudo incluiu todos os indivíduos residentes em Curitiba, Paraná, com diagnóstico confirmado de hepatite A, notificados no Sinan no período de estudo. Foram excluídos indivíduos que não apresentavam confirmação laboratorial por sorologia. Casos graves foram definidos como aqueles que evoluíram para óbito ou necessitaram de transplante hepático em decorrência da infecção pelo vírus da hepatite A.

### Variáveis

No descritivo geral, analisaram-se variáveis sociodemográficas (sexo, idade, raça/cor da pele, escolaridade e local de residência). Na série de casos graves, foram incluídas também informações clínicas (comorbidades, condição de situação de rua, sinais e sintomas, complicações, intervenções terapêuticas e desfecho clínico) e laboratoriais (hemograma, transaminases hepáticas, fosfatase alcalina, bilirrubinas, ureia, creatinina, sódio, potássio e Razão Normalizada Internacional). Foi utilizada a denominação de A a F para descrição dos casos graves e manutenção do anonimato.

### Fontes de dados

Os dados epidemiológicos e demográficos de todos os casos confirmados foram primariamente extraídos do Sinan (notificação de hepatite A). Para a identificação dos desfechos de gravidade, fez-se articulação constante com os NHVE para a confirmação de óbitos e procedimentos de transplante hepático e verificação dos casos informados como óbitos por hepatite A no SIM. Para os casos graves identificados (óbito ou transplante), foi conduzida uma revisão minuciosa de prontuários eletrônicos e físicos, com extração de dados clínicos e laboratoriais, considerando para referência dos dados laboratoriais o dia de maior valor de transaminase glutâmico-oxalacética. Para assegurar a qualidade e consistência dos dados, a coleta de informações-chave foi realizada por meio de planilha validada pela equipe de vigilância local. Para a construção do mapa utilizaram-se os limites geográficos oficiais dos Distritos Sanitários de Curitiba para a representação da distribuição dos casos, de acordo com o endereço deles no Sinan.

### Vieses

As potenciais fontes de viés neste estudo incluem o viés de notificação e o viés de informação. O viés de notificação pode ter ocorrido devido ao não diagnóstico e, consequentemente, subnotificação de casos leves ou assintomáticos de hepatite A no Sinan, limitando a representatividade da totalidade de casos no surto. O viés de informação pode ter ocorrido pelo uso de dados secundários e retrospectivos (Sinan, SIM e prontuários), considerando a potencial incompletude ou heterogeneidade na qualidade dos registros de variáveis sociodemográficas, clínicas e laboratoriais. 

### Tamanho de estudo

O estudo analisou a totalidade dos casos de hepatite A confirmados laboratorialmente, notificados no período analisado (n=281) e a totalidade dos casos classificados como graves (n=6). Não houve cálculo amostral, visto tratar-se de um estudo descritivo de série de casos, que inclui o universo completo dos eventos de interesse.

### Análises estatísticas

Calcularam-se frequências absolutas e relativas, medidas de tendência central (mediana) e dispersão (intervalos interquartílicos) utilizando o Microsoft Excel 2016, o R na interface RStudio (v. 4.4.0) e o QGIS (versão 3.34) para a elaboração do mapa.

## Resultados

Foram identificados, no período analisado, 281 casos confirmados de hepatite A, sendo a maioria (74,7%) do sexo masculino (Tabela 1). A idade mediana dos casos foi de 30 (intervalo interquartílico [IIQ] =13).

**Tabela 1:** Características sociodemográficas dos casos de hepatite A. Curitiba, Paraná, 2023-2024 (n=281)

Características sociodemográficas	Número de casos (%)
Sexo	
Masculino	210 (74,7)
Feminino	71 (25,3)
**Faixa etária (anos)**	
10-19	31 (11,0)
20-29	101 (35,9)
30-39	96 (34,2)
40-49	42 (15,0)
50-59	7 (2,5)
≥60	4 (1,4)
**Raça/cor da pele (n=224)^a^**	
Branca	204 (91,1)
Parda	17 (7,6)
Preta	1 (0,4)
Amarela	1 (0,4)
Indígena	1 (0,4)
**Escolaridade (n=98)^b^**	
1ª a 8ª série incompleta do ensino fundamental	6 (6,1)
Ensino fundamental completo	5 (5,1)
Ensino médio incompleto (antigo colegial ou 2° grau)	17 (17,4)
Ensino médio completo (antigo colegial ou 2° grau)	35 (35,7)
Educação superior incompleta	10 (10,2)
Educação superior completa	25 (25,5)

a,b Percentuais calculados de acordo com o número de indivíduos com informação disponível.

**Figura 1:**
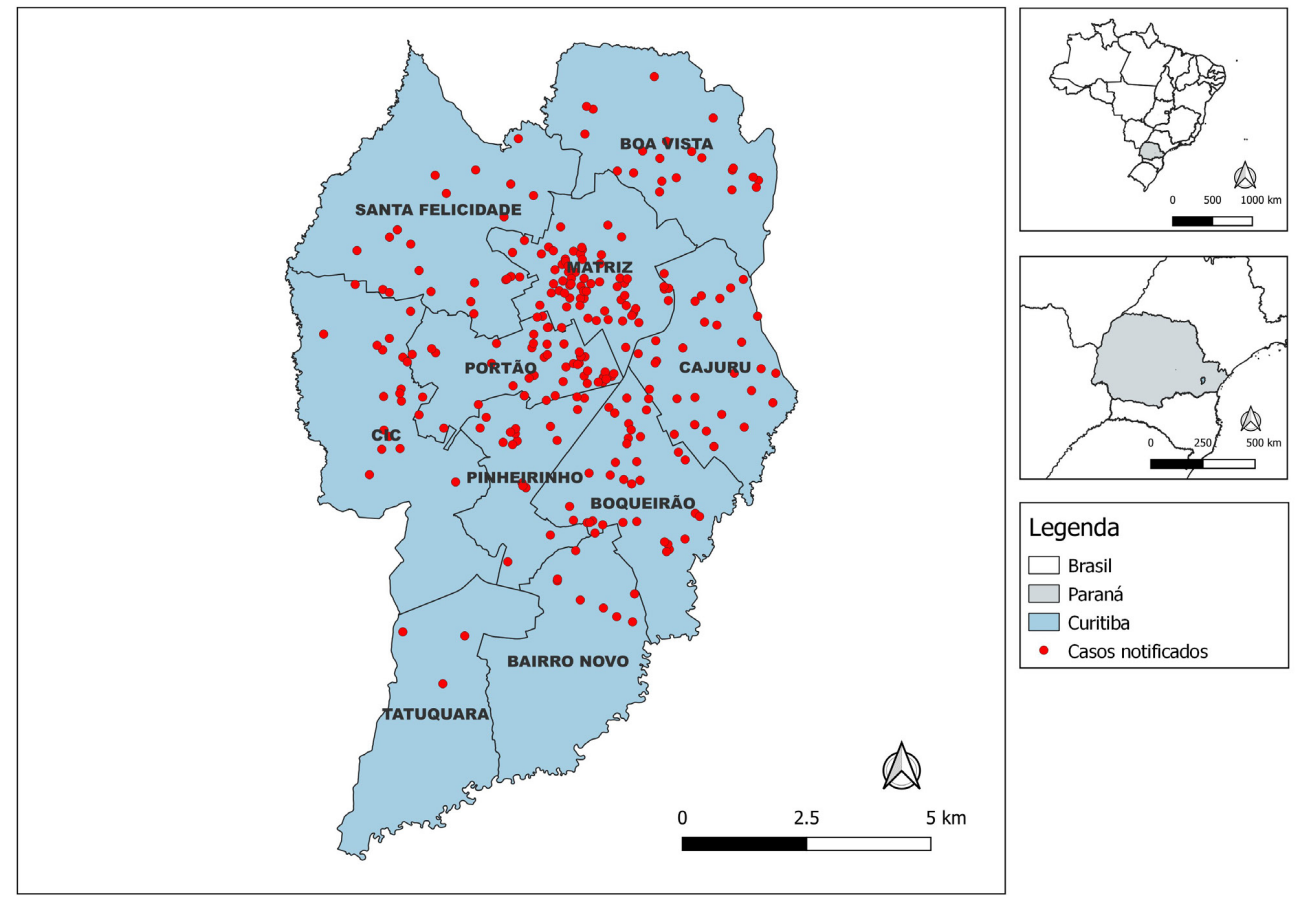
Distribuição dos casos de hepatite A por distrito sanitário. Curitiba, Paraná, 2023-2024 (n=281)

Os distritos sanitários de Matriz (23,0%) e Portão (17,0%) concentraram a maioria dos casos de hepatite A no período de 1 de novembro de 2023 a 29 de maio de 2024 (Figura 1). 

Dos casos confirmados de hepatite A, seis (n=6) foram classificados como casos graves, sendo cinco óbitos e um transplante hepático em decorrência da infecção. Destes seis casos, cinco eram do sexo masculino. Entre esses casos, dois indivíduos encontravam-se em situação de rua no momento da infecção. A mediana de idade foi de 43 anos (IIQ = 13).

Os sintomas mais frequentes foram icterícia (5/6), náuseas e dor abdominal (ambos em 4/6). A principal complicação observada foi insuficiência renal aguda (5/6). Dois casos preencheram critérios clínicos para transplante hepático, tendo o procedimento sido realizado em um deles. Todos os pacientes necessitaram de cuidados em unidade de terapia intensiva. Cinco receberam ventilação mecânica por intubação orotraqueal e transfusão de hemoderivados (Tabela 2). 

**Tabela 2:** Características clínicas e desfechos dos casos graves de hepatite A. Curitiba, Paraná, 2023-2024 (n=6)

Variável/característica^a^	Caso A (40 anos, masculino)	Caso B (61 anos, masculino)	Caso C (29 anos, feminino)	Caso D (39 anos, masculino)	Caso E (49 anos, masculino)	Caso F (54 anos, masculino)
Condições clínicas			
Etilismo	+	-	+	+	-	+
Tabagismo	+	-	+	-	-	+
Diabetes	-	+	-	-	-	+
Dislipidemia	-	-	-	-	-	+
Esteatose hepática	-	+	-	+	+	-
Hepatopatia alcoólica	-	-	-	-	-	+
Transtorno de ansiedade	-	+	+	-	-	-
Doença cardiovascular	-	+	-	+	+	-
Uso de psicoativos	-	-	+	-	-	+
Obesidade	-	-	-	-	+	-
Trombose de MMII^a^	-	-	-	-	+	-
**Sintomas**			
Náuseas	+	+	+	-	+	-
Vômitos	+	+	+	-	-	-
Inapetência	+	+	-	+	-	-
Dor abdominal	+	-	+	+	+	-
Fadiga	+	-	+	-	-	-
Colúria	+	-	+	-	+	-
Icterícia	+	-	+	+	+	+
Esclera ictérica	+	-	-	-	+	+
Fraqueza	+	-	-	-	-	-
Febre	-	-	+	-	+	-
Mialgia	-	-	+	+	-	-
Artralgia	-	-	+	-	-	-
Diarreia	-	-	+	-	-	-
Prurido	-	-	+	-	-	-
Calafrios	-	-	+	-	-	-
Disúria	-	-	+	-	-	-
Hepatomegalia	-	-	-	+	-	+
Dor em MMII^b^	-	-	-	+	-	-
Esplenomegalia	-	-	-	-	+	-
Hemoptise	-	-	-	-	+	-
Acolia fecal	-	-	-	-	+	-
Edema MMII^b^	-	-	-	-	-	+
Cansaço	-	-	-	-	-	+
Dispneia	-	-	-	-	-	+
Perda de peso	-	-	-	-	-	+
**Complicações**			
Acidose metabólica	+	+	+	+	-	-
Choque séptico	+	-	-	-	-	-
Encefalopatia hepática	+	-	-	-	+	-
Hepatite fulminante	+	-	+	-	+	-
Hipoalbuminemia	+	-	-	-	-	+
Hipocalemia severa	+	-	-	-	-	-
Insuficiência renal aguda	+	+	+	+	+	-
SARA^c^	+	-	-	-	-	-
Insuficiência hepática aguda	-	+	+	-	-	-
Melena	-	+	-	-	-	-
Síndrome hepatorrenal	-	+	-	-	-	-
Hiperlactatemia	-	-	+	-	-	-
AVC^d^ isquêmico	-	-	-	+	-	-
Coagulopatia	-	-	-	-	-	+
Hipocalemia	-	-	-	-	+	-
Hiponatremia	-	-	-	-	+	-
Hepatite alcoólica agudizada	-	-	-	+	-	-
Tacrolinemia	-	-	-	-	+	-
**Necessidades terapêuticas**			
Ventilação mecânica	+	+	+	+	+	SI^e^
Transfusão de hemoderivados	+	+	+	+	+	SI^e^
Hemodiálise	-	+	+	+	-	SI^e^
**Desfecho**	Óbito	Óbito	Óbito	Óbito	Transplante hepático	Óbito

a + indica presença da variável; - indica ausência da variável; ^b^ membros inferiores; ^c^ síndrome da angústia respiratória aguda; ^d^ Acidente Vascular Cerebral; ^e^ sem informações.

A duração da internação hospitalar variou de 6 a 41 dias, com mediana de 11 dias. Todos os óbitos ocorreram durante a hospitalização (Tabela 3).

As enzimas hepáticas, especificamente as transaminases glutâmico-oxalacética e transaminase glutâmico-pirúvica estavam acima do limite de referência em mais de cem vezes. O comprometimento sistêmico foi confirmado pela Razão Normalizada Internacional mediana três vezes o valor de referência e pela hiperbilirrubinemia acentuada. Os resultados laboratoriais incluem disfunção renal aguda (creatinina mediana de 3 mg/dL [IIQ=5]) e alterações hematológicas como leucocitose e trombocitopenia (Tabela 4).

**Tabela 3:** Condição de situação de rua, mediana e intervalo interquartílico (IIQ) entre eventos clínicos em casos graves de hepatite A. Curitiba, Paraná, 2023-2024 (n=6)

Caso	Situação de rua^a^	Início sintomas até 1º atendimento (dias)	1º atendimento até internação hospitalar (dias)	Internação hospitalar até desfecho (dias)
A, 40 anos, masculino	+	7	0	11
B, 61 anos, masculino	-	0	6	11
C, 29 anos, feminino	-	7	4	6
D, 39 anos, masculino	+	7	0	33
E, 49 anos, masculino	-	7	21	41
F, 54 anos, masculino	-	11	6	9
Mediana (IIQ)		7 (0)	5 (5)	11 (18)

a + indica presença da variável; - indica ausência da variável.

**Tabela 4:** Mediana, primeiro (Q1) e terceiro quartis (Q3) dos parâmetros laboratoriais dos casos graves de hepatite A. Curitiba, Paraná, 2023-2024 (n=6)

Parâmetro laboratorial	Mediana (Q1; Q3)^b^	Referência laboratorial
Hemoglobina (g/dL)	10 (10; 14)	13,5-17,5
Leucócitos totais (/mm^3^)	13.095 (5.938; 21.648)	3.800-11.000
Linfócitos (/mm^3^)	1.530 (1.010; 2.337)	760-4.950
Plaquetas (/mm^3^)	112.500 (97.675; 161.750)	150.000-450.000
Transaminase glutâmico-oxalacética (U/L)	3.448 (2.092; 4.504)	5-34
Transaminase glutâmico-pirúvica (U/L)	1.948 (472; 4.525)	<45
Gama glutamil transferase (U/L)	237 (191; 292)	12-64
Fosfatase alcalina (U/L)	209 (175; 310)	50-116
Ureia (mg/dL)	52 (23; 102)	18-55
Creatinina (mg/dL)	3 (1; 6)	0,72-1,25
Sódio (mmol/L)	130 (127; 135)	136-145
Potássio (mmol/L)	4 (3; 6)	3,5-5,1
Bilirrubina total (mg/dL)	16 (13; 18)	0,2-1,2
Bilirrubina direta (mg/dL)	12 (10; 13)	0-0,5
Bilirrubina indireta (mg/dL)	4 (3; 5)	<1,1
Razão Normalizada Internacional^a^	3 (2; 4)	≤1

a valor de referência para pessoas sem uso de medicações anticoagulantes; ^b^ medidas de tendência central e dispersão calculada para o total de indivíduos com informação disponível.

## Discussão

O surto de hepatite A em Curitiba apresentou predominância entre adultos jovens do sexo masculino, com concentração territorial em distritos centrais bem estruturados e com ocorrência de casos que evoluíram para formas graves. Os casos graves caracterizaram-se por disfunção hepática e renal importantes, elevada frequência de complicações e necessidade de suporte intensivo, culminando em desfechos desfavoráveis - transplante e óbito. Esses achados reforçam que, embora geralmente autolimitada, a hepatite A pode evoluir de forma abrupta e severa. 

Entre as limitações do estudo, destacam-se o uso de dados secundários e a restrição a informações como exames laboratoriais, o que impediu o acompanhamento da evolução dos parâmetros clínicos ao longo da internação. A possibilidade de subnotificação e o uso de dados secundários, sujeitos a subnotificação, incompletude e heterogeneidade de qualidade entre sistemas e prontuários também deve ser considerada na interpretação dos resultados. 

A mediana de idade dos casos graves foi superior à mediana observada no surto geral, sugerindo que a idade avançada pode ter contribuído para a evolução desfavorável da infecção. O predomínio do sexo masculino entre os casos graves acompanha a distribuição sociodemográfica do surto geral, mas é mais expressivo do que a predominância registrada nos dados nacionais de 2012-2022, nos quais os casos estavam distribuídos entre 53,8% de homens e 46,2% de mulheres (10). Em 2022, essa diferença aumentou, com 64,0% dos casos em homens, frequentemente relacionados a redes comportamentais específicas e maior exposição a práticas de risco (11-12-13). Tais evidências ressaltam a importância de estratégias direcionadas, como ações de educação em saúde tanto para os riscos de transmissão por alimentos ou água contaminados como via contato sexual e ampliação da cobertura vacinal para grupos sob maior risco em contextos epidêmicos, em consonância com a literatura (14,15). 

O predomínio da raça/cor da pele branca reflete a composição demográfica do município (16). A concentração de casos nos distritos sanitários da região norte do município, como Matriz e Portão, situados em áreas mais desenvolvidas e centrais da cidade, sugere uma transmissão localizada em contextos urbanos com melhor infraestrutura, contrastando com o padrão classicamente associado a condições de maior vulnerabilidade socioeconômica e saneamento precário (3,17). Tal padrão reforça a hipótese de envolvimento de fatores comportamentais e redes de contato específicas na dinâmica de transmissão.

A proporção de casos que evoluíram para óbito é levemente superior ao padrão (<1%) descrito na literatura (2), sublinhando a vulnerabilidade da população. A ocorrência de hepatite fulminante, embora rara, é reconhecidamente mais frequente em adultos e na presença de comorbidades, como hepatopatia alcoólica e esteatose hepática (4). Esses achados reforçam o potencial de gravidade da infecção pelo vírus da hepatite A em contextos clínicos e epidemiológicos específicos e sublinham a necessidade de uma resposta assistencial estruturada, com foco na detecção precoce de casos graves e no acesso oportuno a cuidados especializados durante surtos.

A análise cronológica dos eventos clínicos evidencia a natureza frequentemente insidiosa da progressão da hepatite A para formas graves em adultos. O intervalo prolongado entre o início dos sintomas e o primeiro atendimento pode resultar de uma baixa percepção da gravidade. A ampla variação no tempo decorrido até a hospitalização reflete a heterogeneidade da apresentação clínica e a necessidade de um elevado índice de suspeição para casos que evoluem rapidamente. A presença de dois indivíduos em situação de rua entre os casos graves reforça a associação entre vulnerabilidade social e desfechos adversos. Essa associação tem sido descrita em surtos urbanos, e sustenta a recomendação de inclusão de populações socialmente vulneráveis nas estratégias de imunização (15). 

As manifestações observadas entre os casos graves - icterícia, insuficiência renal aguda, encefalopatia e necessidade de suporte intensivo - são compatíveis com os padrões descritos em quadros graves de hepatite A (2). A elevação expressiva de transaminases, bilirrubinas e razão normalizada internacional, aliada ao comprometimento renal, sugere evolução para síndrome hepatorrenal em parte dos casos, condição associada na literatura a prognóstico reservado (18,19). Esse padrão laboratorial reforça a necessidade de reconhecimento precoce e manejo especializado em contexto hospitalar. 

O surto de hepatite A em Curitiba evidenciou que, mesmo em áreas com infraestrutura sanitária consolidada, a infecção pode evoluir para formas graves, com alta complexidade clínica e risco de desfechos fatais. Os achados destacam a importância da vigilância sensível, do reconhecimento precoce de sinais de gravidade, da ampliação da cobertura vacinal e da articulação entre atenção básica, vigilância e serviços hospitalares como estratégias centrais para reduzir morbimortalidade em cenários epidêmicos. 

## Data Availability

Os bancos de dados anonimizados utilizados nesta pesquisa encontram-se disponíveis publicamente no repositório SciELO Data, associado à revista Epidemiologia e Serviços de Saúde (RESS), podendo ser acessados por meio do link:https://data.scielo.org/dataset.xhtml?persistentId=doi:10.48331/SCIELODATA.CDJTUW. O conjunto de dados deve ser citado de acordo com a referência indicada no próprio repositório.

## References

[1] Feinstone SM, Kapikian AZ, Purcell RH (1973). Hepatitis A: detection by immune electron microscopy of a viruslike antigen associated with acute illness. Science.

[2] Lai M., Chopra S (2024). Hepatitis A virus infection in adults: epidemiology, clinical manifestations, and diagnosis [Internet].

[3] World Health Organization (2023). Hepatitis A: key facts [Internet].

[4] Hofmeister MG, Xing J, Foster MA, Augustine RJ, Burkholder C, Collins J (2021). Factors associated with hepatitis A mortality during person-to-person outbreaks: a matched case-control study-United States, 2016-2019. Hepatology.

[5] Jacobsen KH (2018). Globalization and the changing epidemiology of hepatitis A virus. Cold Spring Harb Perspect Med.

[6] Madalosso G, Kamioka GA, Bassit NP, Pavanello EI, Sousa SCZ, Koizumi IK (2018). Surto de hepatite A em homens que fazem sexo com homens no município de São Paulo, Brasil, 2017. Braz J Infect Dis.

[7] Prefeitura de Curitiba (2025). Surto de hepatite A em Curitiba tem transmissão de pessoa a pessoa: são 255 casos e 5 mortes confirmadas em 2024 [Internet].

[8] Shin EC, Jeong SH (2018). Natural history, clinical manifestations, and pathogenesis of hepatitis A. Cold Spring Harb Perspect Med.

[9] Instituto Brasileiro de Geografia e Estatística (2022). Censo 2022 [Internet].

[10] Ministério da Saúde (BR) (2023). Boletim epidemiológico: hepatites virais 2023 [Internet].

[11] Nicolay N, Le Bourhis-Zaimi M, Lesourd A, Martel M, Roque-Afonso AM, Erouart S (2020). Description of a hepatitis A outbreak in men who have sex with men, Seine-Maritime, France, 2017. BMC Public Health.

[12] Ndumbi P, Freidl GS, Williams CJ, Mårdh O, Varela C, Avellón A (2018). Hepatitis A outbreak disproportionately affecting men who have sex with men in the European Union and European Economic Area, 2016-2017. Euro Surveill.

[13] Rosendal E, von Schreeb S, Gomes A, Lino S, Grau-Pujol B, Magalhães S (2024). Ongoing outbreak of hepatitis A associated with sexual transmission among men who have sex with men, Portugal, 2023-2024. Euro Surveill.

[14] Castro LS, Rezende GR, Pires Fernandes FR, Bandeira LM, Cesar GA, Lago BV (2021). HAV infection in Brazilian men who have sex with men: the importance of surveillance to avoid outbreaks. PLoS One.

[15] Hennessey KA, Bangsberg DR, Weinbaum C, Hahn JA (2009). Hepatitis A seroprevalence and risk factors among homeless adults in San Francisco. Public Health Rep.

[16] Instituto Brasileiro de Geografia e Estatística (2024). Censo demográfico 2022: panorama [Internet].

[17] Carrilho FJ, Mendes Clemente C, Silva LC (2005). Epidemiology of hepatitis A and E virus infection in Brazil. Gastroenterol Hepatol.

[18] Choi HK, Song YG, Han SH, Ku NS, Jeong SJ, Baek JH (2011). Clinical features and outcomes of acute kidney injury among patients with acute hepatitis A. J Clin Virol.

[19] Andrievskaya M, Lenhart A, Uduman J (2019). Emerging threat: changing epidemiology of hepatitis A and acute kidney injury. Adv Chronic Kidney Dis.

