# Supplementary material for: Casos graves de hepatite A: série de casos em Curitiba, Paraná, 2023-2024
Source: Epidemiol Serv Saude. 2026 Mar 27;35:e20250869. [Article in Portuguese] doi: 10.1590/S2237-96222026v35e20250869.pt (PMC13035272; doi:10.1590/S2237-96222026v35e20250869.pt)
Supplement: Translation

# Translation: Severe cases of hepatitis A: case series in Curitiba, Paraná, 2023-2024

**DOI:** 10.1590/S2237-96222026v35e20250869.en

**Authors:** Janilza Silveira Silva, Renata Barbosa Vilaça Marques de Carvalho, Alcides Souto de Oliveira, Liza Regina Bueno Rosso, Leia Regina Silva, Diego Spinoza dos Santos, Clea Elisa Ribeiro, Katiuska Ferraz Jansen Negrello, Cláudia Weingaertner Palm, Dirlene Pacheco Venâncio, Monique Boese, Aroldo José Borges Carneiro, Tatiane Bartneck Telles, Silvio Luis Rodrigues Almeida

**Affiliations:** 1 Ministério da Saúde, Departamento de Emergências em Saúde Pública, Brasília, DF, Brazil; 2 Centro de Epidemiologia da Secretaria Municipal da Saúde de Curitiba, PR, Brazil; 3 Centro de Informações Estratégicas em Vigilância em Saúde de Curitiba, PR, Brazil; 4 Prefeitura de Curitiba, Secretaria Municipal de Saúde, Curitiba, PR, Brazil

**Keywords:** Hepatitis, Hepatitis A Virus, Acute Liver Failure, Liver Failure, Descriptive Studies.

## Abstract

**Objective:**

Describe the epidemiological profile of the hepatitis A outbreak in Curitiba, Paraná, between November 2023 and May 2024, with emphasis on the clinical and laboratory characterization of cases that progressed to death or liver transplantation.

**Methods:**

Descriptive case series study based on confirmed cases of hepatitis A. Severe cases, defined by death or liver transplantation, were identified via the Mortality Information System and Hospital Epidemiological Surveillance Centers. Clinical and laboratory data were obtained by reviewing medical records. Proportions, measures of central tendency, and dispersion were calculated.

**Results:**

Among the 281 confirmed cases, the majority were male (74.7%), with a median age of 29 years. Six people (2.1%) progressed to severe forms, of whom three received a diagnosis of fulminant hepatitis. The median age of the severe cases was 43 years, with a male predominance (5/6), and two were homeless individuals. Among the severe cases, five resulted in death, and one in a liver transplant. The most common symptoms and complications were jaundice, acute renal failure, and the need for mechanical ventilation (5/6). Laboratory results indicated hepatic dysfunction (median aspartate aminotransferase of 3,448 U/L; median international normalized ratio of 3) and renal dysfunction (median urea 52 mg/dL and creatinine 3 mg/dL). The median hospitalization length was 11 days.

**Conclusion:**

In this outbreak, there were cases requiring liver transplantation and progressing to death, demonstrating that, although generally self-limiting, the disease can present with a severe course. Timely response and targeted vaccination may be key strategies in outbreak scenarios.

**Ethical aspects**

**This research respected the ethical principles, having obtained the following approval data:**

Research Ethics Committee: National Ethics Committee for Research

Opinion number: 7,545,013 

Approval date: 13/6/2025

Certificate of Submission for Ethical Appraisal: 87956525.0.0000.0008

Informed Consent Form: Not applicable.

## Introduction

Hepatitis A is an acute infectious liver disease caused by the hepatitis A virus, a nonenveloped, single-stranded RNA picornavirus belonging to the genus *Hepatovirus* (1,2). Its transmission occurs mainly via the fecal-oral route, through the consumption of contaminated water or food, as well as via direct contact (oro-anal) with infected people (3).

While most cases are self-limiting and resolve spontaneously, especially among children, the infection can progress to severe forms, particularly in adults or individuals with comorbidities (4). In these cases, fulminant hepatitis can occur - characterized by acute liver failure, encephalopathy, and coagulopathy - in addition to systemic complications such as acute renal failure, sepsis, and metabolic disorders, often requiring intensive care and liver transplantation as a rescue measure (2).

In recent decades, a transition in the epidemiological pattern of hepatitis A has been observed in several countries (5). Improved sanitation conditions have reduced early exposure to the virus, increasing susceptibility among adolescents and young adults, age groups in which infection can be more symptomatic and severe (5). In Brazil, this transition is also manifested with localized outbreaks, often affecting specific populations, such as men who have sex with men (6).

Between November 2023 and May 2024, the municipality of Curitiba, Paraná, Brazil, experienced an outbreak of hepatitis A with deaths (7). The viral circulation of hepatitis A in localized outbreaks, such as the one recorded in Curitiba, poses a significant risk to the adult population, which is more susceptible to severe forms of the disease (8). The potential occurrence of unfavorable clinical outcomes, such as fulminant hepatitis, requires early recognition of clinical and epidemiological risk factors to mitigate mortality. The characterization of severe cases is therefore essential to support surveillance, optimize clinical management, and strengthen the public health response. 

The study aimed to describe the epidemiological profile of the hepatitis A outbreak in Curitiba, Paraná, between November 2023 and May 2024, with emphasis on the clinical and laboratory characterization of cases that progressed to death or liver transplantation.

## Methods

### Design

This is a descriptive case series study of hepatitis A, with emphasis on severity outcomes, confirmed in Curitiba, Paraná, Brazil, between November 1, 2023, and May 29, 2024.

### Context

Curitiba, Paraná, has a resident population of 1,773,718 inhabitants (9). This study was conducted during a hepatitis A outbreak, with 281 confirmed cases reported from November 1, 2023, to May 29, 2024. The municipal epidemiological surveillance system notifies hepatitis A cases in the Notifiable Diseases Information System (SINAN). It monitors outbreak severity using data from the Mortality Information System (SIM) and coordination with the Hospital Epidemiological Surveillance Centers (NHVE). 

This study was conducted by a team from the Training Program in Epidemiology Applied to the Unified Health System Services (EpiSUS-Avançado), in partnership with the local surveillance team, to ensure the findings could be readily applied to the outbreak response. It originated from an urgent epidemiological surveillance action at the invitation of the municipality.

### Participants

The study included all individuals residing in Curitiba, Paraná, with a confirmed hepatitis A diagnosis reported in the SINAN during the study period. Individuals without laboratory confirmation by serology were excluded. Severe cases were defined as those that progressed to death or required liver transplantation as a result of infection with the hepatitis A virus.

### Variables

In the general description, sociodemographic variables (sex, age, race/skin color, education, and place of residence) were analyzed. In the series of severe cases, clinical information (comorbidities, homelessness status, signs and symptoms, complications, therapeutic interventions, and clinical outcome) and laboratory information (blood count, liver transaminases, alkaline phosphatase, bilirubin, urea, creatinine, sodium, potassium, and International Normalized Ratio) were also included. The designation A to F was used to describe severe cases and maintain anonymity.

### Data sources

Epidemiological and demographic data for all confirmed cases were primarily extracted from SINAN (hepatitis A notifications). To identify severity outcomes, constant coordination was made with the NHVE to confirm deaths and liver transplant procedures, and to verify cases reported as deaths due to hepatitis A in SIM. For identified severe cases (death or transplant), a thorough review of electronic and physical medical records was conducted, extracting clinical and laboratory data, and using the day with the highest glutamic-oxaloacetic transaminase value as the reference for laboratory data. To ensure data quality and consistency, key information was collected using a spreadsheet validated by the local surveillance team. The official geographic boundaries of the Curitiba Health Districts were used to represent the distribution of cases, based on their addresses in the SINAN database.

### Biases

Potential sources of bias in this study include reporting bias and information bias. Reporting bias may have occurred due to non-diagnosis and, consequently, underreporting of mild or asymptomatic hepatitis A cases in SINAN, limiting the representativeness of the total number of cases in the outbreak. Information bias may have occurred due to the use of secondary and retrospective data (SINAN, SIM, and medical records), considering the potential incompleteness or heterogeneity in the quality of records of sociodemographic, clinical, and laboratory variables. 

### Study size

The study analyzed all laboratory-confirmed hepatitis A cases reported during the study period (n=281) and all severe cases (n=6). No sample size calculation was performed because this is a descriptive case series study that includes the complete universe of events of interest.

### Statistical analyses

Absolute and relative frequencies, measures of central tendency (median), and dispersion (interquartile ranges) were calculated using Microsoft Excel 2016, R in the RStudio interface (v. 4.4.0), and QGIS (version 3.34) for map creation.

### Results

During the analyzed period, 281 confirmed cases of hepatitis A were identified, the majority (74.7%) being male (Table 1). The median age of the cases was 30 (interquartile range [IQR] =13).

**Table 1:** Sociodemographic characteristics of hepatitis A cases. Curitiba, Paraná, 2023-2024 (n=281)

Sociodemographic characteristics	Number of cases (%)
Sex	
Male	210 (74.7)
Female	71 (25.3)
**Age range (years)**	
10-19	31 (11.0)
20-29	101 (35.9)
30-39	96 (34.2)
40-49	42 (15.0)
50-59	7 (2.5)
≥60	4 (1.4)
**Race/skin color (n=224)^a^**	
White	204 (91.1)
Mixed-race	17 (7.6)
Black	1 (0.4)
Yellow	1 (0.4)
Indigenous	1 (0.4)
**Education (n=98)^b^**	
Incomplete 1^st^ to 8^th^ grade of elementary and middle school	6 (6.1)
Complete middle school	5 (5.1)
Incomplete high school	17 (17.4)
Complete high school	35 (35.7)
Incomplete higher education	10 (10.2)
Complete higher education	25 (25.5)

a,b Percentages calculated based on the number of individuals with available information.

The health districts of Matriz (23.0%) and Portão (17.0%) accounted for the majority of hepatitis A cases during the period from November 1, 2023, to May 29, 2024 (Figure 1). 

**Figure 1:** Distribution of hepatitis A cases by health district. Curitiba, Paraná, 2023-2024 (n=281)

Of the confirmed cases of hepatitis A, six (n=6) were classified as severe cases, with five deaths and one liver transplant due to the infection. Of these six cases, five were male. Among these cases, two individuals were homeless at the time of infection. The median age was 43 years (IQR = 13).

The most frequent symptoms were jaundice (5/6), nausea, and abdominal pain (both in 4/6). The main complication observed was acute renal failure (5/6). Two cases met clinical criteria for liver transplantation, and the procedure was performed in one of them. All patients required intensive care unit care. Five received mechanical ventilation via orotracheal intubation and blood product transfusions (Table 2).

**Table 2:** Clinical characteristics and outcomes of severe cases of hepatitis A. Curitiba, Paraná, 2023-2024 (n=6)

Characteristic^a^	Case A (40 years, male)	Case B (61 years, male)	Case C (29 years, female)	Case D (39 years, male)	Case E (49 years, male)	Case F (54 years, male)
Clinical conditions			
Alcoholism	+	-	+	+	-	+
Smoking	+	-	+	-	-	+
Diabetes	-	+	-	-	-	+
Dyslipidemia	-	-	-	-	-	+
Hepatic steatosis	-	+	-	+	+	-
Alcoholic liver disease	-	-	-	-	-	+
Anxiety disorder	-	+	+	-	-	-
Cardiovascular disease	-	+	-	+	+	-
Psychoactive substance use	-	-	+	-	-	+
Obesity	-	-	-	-	+	-
Lower limb thrombosis	-	-	-	-	+	-
**Symptoms**			
Nausea	+	+	+	-	+	-
Vomiting	+	+	+	-	-	-
Loss of appetite	+	+	-	+	-	-
Abdominal pain	+	-	+	+	+	-
Fatigue	+	-	+	-	-	-
Choluria	+	-	+	-	+	-
Jaundice	+	-	+	+	+	+
Scleral icterus	+	-	-	-	+	+
Weakness	+	-	-	-	-	-
Fever	-	-	+	-	+	-
Myalgia	-	-	+	+	-	-
Arthralgia	-	-	+	-	-	-
Diarrhea	-	-	+	-	-	-
Pruritus	-	-	+	-	-	-
Chills	-	-	+	-	-	-
Dysuria	-	-	+	-	-	-
Hepatomegaly	-	-	-	+	-	+
Lower limb pain	-	-	-	+	-	-
Splenomegaly	-	-	-	-	+	-
Hemoptysis	-	-	-	-	+	-
Acholic stools	-	-	-	-	+	-
Lower limb edema	-	-	-	-	-	+
Tiredness	-	-	-	-	-	+
Dyspnea	-	-	-	-	-	+
Weight loss	-	-	-	-	-	+
**Complications**			
Metabolic acidosis	+	+	+	+	-	-
Septic shock	+	-	-	-	-	-
Hepatic encephalopathy	+	-	-	-	+	-
Fulminant hepatitis	+	-	+	-	+	-
Hypoalbuminemia	+	-	-	-	-	+
Severe hypokalemia	+	-	-	-	-	-
Acute renal failure	+	+	+	+	+	-
ARDS^b^	+	-	-	-	-	-
Acute liver failure	-	+	+	-	-	-
Melena	-	+	-	-	-	-
Hepatorenal syndrome	-	+	-	-	-	-
Hyperlactatemia	-	-	+	-	-	-
Ischemic stroke	-	-	-	+	-	-
Coagulopathy	-	-	-	-	-	+
Hypokalemia	-	-	-	-	+	-
Hyponatremia	-	-	-	-	+	-
Acute alcoholic hepatitis	-	-	-	+	-	-
Elevated Tacrolimus levels	-	-	-	-	+	-
**Therapeutic needs**			
Mechanical ventilation	+	+	+	+	+	NI^c^
Blood product transfusion	+	+	+	+	+	NI^c^
Hemodialysis	-	+	+	+	-	NI^c^
**Outcome**	Death	Death	Death	Death	Liver transplant	Death

a + indicates the presence of the variable; - indicates the absence of the variable; ^b^ acute respiratory distress syndrome; ^c^no information.

The length of hospital stay ranged from 6 to 41 days, with a median of 11 days. All deaths occurred during hospitalization (Table 3).

Liver enzymes, specifically glutamic-oxaloacetic transaminase and glutamic-pyruvic transaminase, were more than a hundredfold above the reference limit. Systemic involvement was confirmed by a median International Normalized Ratio three times the reference value and marked hyperbilirubinemia. Laboratory results included acute renal dysfunction (median creatinine of 3 mg/dL [IQR=5]) and hematological abnormalities such as leukocytosis and thrombocytopenia (Table 4).

**Table 3:** Homelessness status, median and interquartile range (IQR) between clinical events in severe cases of hepatitis A. Curitiba, Paraná, 2023-2024 (n=6)

Case	Homelessness^a^	Onset of symptoms until 1^st^ medical attention (days)	1^st^ medical attention until hospital admission (days)	Hospital admission until outcome (days)
A, 40 years, male	+	7	0	11
B, 61 years, male	-	0	6	11
C, 29 years, female	-	7	4	6
D, 39 years, male	+	7	0	33
E, 49 years, male	-	7	21	41
F, 54 years, male	-	11	6	9
Median (IQR)		7 (0)	5 (5)	11 (18)

a + indicates the presence of the variable; - indicates the absence of the variable.

**Table 4:** Median, first (Q1) and third quartiles (Q3) of laboratory parameters of severe cases of hepatitis A. Curitiba, Paraná, 2023-2024 (n=6)

Laboratory parameter	Median (Q1; Q3)^b^	Laboratory reference
Hemoglobin (g/dL)	10 (10; 14)	13.5-17.5
Total leukocytes (/mm^3^)	13,095 (5,938; 21,648)	3,800-11,000
Lymphocytes (/mm^3^)	1,530 (1,010; 2,337)	760-4,950
Platelets (/mm^3^)	112,500 (97,675; 161,750)	150,000-450,000
Aspartate aminotransferase (U/L)	3,448 (2,092; 4,504)	5-34
Alanine aminotransferase (U/L)	1,948 (472; 4,525)	<45
Gamma glutamyl transferase (U/L)	237 (191; 292)	12-64
Alkaline phosphatase (U/L)	209 (175; 310)	50-116
Urea (mg/dL)	52 (23; 102)	18-55
Creatinine (mg/dL)	3 (1; 6)	0.72-1.25
Sodium (mmol/L)	130 (127; 135)	136-145
Potassium (mmol/L)	4 (3; 6)	3.5-5.1
Total bilirubin (mg/dL)	16 (13; 18)	0.2-1.2
Direct bilirubin (mg/dL)	12 (10; 13)	0-0.5
Indirect bilirubin (mg/dL)	4 (3; 5)	< 1.1
International Normalized Ratio^a^	3 (2; 4)	≤ 1

a reference value for people not using anticoagulant medications; ^b^ measures of central tendency and dispersion calculated for the total number of individuals with available information.

## Discussion

The hepatitis A outbreak in Curitiba showed a predominance among young adult males, with territorial concentration in well-structured central districts, and with cases that progressed to severe forms. Severe cases were characterized by significant hepatic and renal dysfunction, a high frequency of complications, and the need for intensive support, culminating in unfavorable outcomes - transplantation and death. These findings reinforce that, although generally self-limiting, hepatitis A can progress abruptly and severely. 

Among the study limitations, the use of secondary data and the restriction to information such as laboratory tests stand out, which prevented the monitoring of the evolution of clinical parameters throughout hospitalization. The possibility of underreporting and the use of secondary data, which are subject to underreporting, incompleteness, and heterogeneity in quality across systems and medical records, should also be considered when interpreting the results. 

The median age of severe cases was higher than the median observed in the overall outbreak, suggesting that advanced age may have contributed to a more unfavorable course of infection. The predominance of males among severe cases follows the sociodemographic distribution of the overall outbreak. However, it is more pronounced than the predominance recorded in the national data from 2012-2022, in which cases were distributed between 53.8% men and 46.2% women (10). In 2022, this difference increased, with 64.0% of cases in men, often associated with specific behavioral networks and greater exposure to risky practices (11-12-13). Such evidence highlights the importance of targeted strategies, such as health education actions on the risks of transmission through contaminated food or water and via sexual contact, and expanding vaccination coverage for groups at higher risk in epidemic contexts, in line with the literature (14,15). 

The predominance of the white race/skin color reflects the municipality’s demographic composition (16). The concentration of cases in the health districts of the municipality’s northern region, such as Matriz and Portão, located in more developed and central areas of the city, suggests localized transmission in urban contexts with better infrastructure, contrasting with the pattern classically associated with conditions of greater socioeconomic vulnerability and poor sanitation (3,17). This pattern reinforces the hypothesis that behavioral factors and specific contact networks are involved in transmission dynamics.

The proportion of cases that progressed to death is slightly higher than the standard (<1%) described in the literature (2), underlining the vulnerability of the population. The occurrence of fulminant hepatitis, although rare, is recognized as more frequent in adults and in the presence of comorbidities, such as alcoholic liver disease and hepatic steatosis (4). These findings reinforce the potential severity of hepatitis A virus infection in specific clinical and epidemiological contexts and underscore the need for a structured care response that focuses on early detection of severe cases and timely access to specialized care during outbreaks.

The chronological analysis of clinical events highlights the often insidious nature of the progression of hepatitis A to severe forms in adults. The prolonged interval between symptom onset and first medical attention may result from a low perceived severity. The wide variation in the time to hospitalization reflects the heterogeneity of clinical presentation and the need for a high index of suspicion in rapidly progressing cases. The presence of two homeless individuals among the severe cases reinforces the association between social vulnerability and adverse outcomes. This association has been described in urban outbreaks and supports the recommendation to include socially vulnerable populations in immunization strategies (15). 

The manifestations observed among the severe cases - jaundice, acute renal failure, encephalopathy, and the need for intensive support - are consistent with the patterns described in severe cases of hepatitis A (2). The significant elevation of transaminases, bilirubin, and international normalized ratio, coupled with renal impairment, suggests progression to hepatorenal syndrome in some cases, a condition associated in the literature with a guarded prognosis (18,19). This laboratory pattern reinforces the need for early recognition and specialized management in a hospital setting. 

The hepatitis A outbreak in Curitiba highlighted that, even in areas with consolidated health infrastructure, the infection can progress to severe forms, with high clinical complexity and risk of fatal outcomes. The findings emphasize the importance of sensitive surveillance, early recognition of severity, expansion of vaccination coverage, and coordination among primary care, surveillance, and hospital services as central strategies to reduce morbidity and mortality in epidemic scenarios. 

## Data Availability

The anonymized databases used in this study are publicly available in the SciELO Datarepository, linked to the journal Epidemiologia e Serviços de Saúde (RESS), and can be accessed at:https://data.scielo.org/dataset.xhtml?persistentId=doi:10.48331/SCIELODATA.CDJTUW. The dataset should be cited according to the reference provided in the repository.
